# Coeliac disease in a child with anorectal malformation: The importance of considering other causes of diarrhea

**DOI:** 10.4103/0971-9261.69140

**Published:** 2010

**Authors:** Milan Gopal, Shawqui Nour, Wren Hoskyns

**Affiliations:** Department of Paediatric Surgery, Leicester Royal Infirmary, Infirmary Square, Leicester, LE1 5WW, UK; 1 Department of General Pediatrics, Leicester Royal Infirmary, Infirmary Square, Leicester, LE1 5WW, UK

**Keywords:** Anorectal malformation, coeliac disease, fecal incontinence

## Abstract

We present the case of an Indian child with a high anorectal malformation (ARM) who postoperatively had troublesome fecal incontinence. Based on a dietary history, weight loss, and diarrhea, a duodenal biopsy was performed that revealed coeliac disease. Since being on a gluten-free diet, her symptoms have improved dramatically. To the best of our knowledge this is the first report in the English literature of such an association between ARMs and coeliac disease. Dietary modification alone can dramatically improve symptoms in these children.

## INTRODUCTION

Coeliac disease was traditionally thought to be a disease of the western hemisphere. However, recently there has been a large increase in the diagnosis of this condition in the East, particularly in North India, where wheat is a staple part of the diet. We present the case of a child of Indian origin living in the UK who was born with an anorectal malformation (ARM), in whom the subsequent diagnosis of coeliac disease significantly altered her management.

## CASE REPORT

The patient is a female of Indian origin born at term of a nonconsanguineous marriage. The antenatal period was normal and there is no family history of coeliac disease. At birth she was noted to have an imperforate anus with no obvious fistula. There were no features suggestive of Down’s syndrome. A loop sigmoid colostomy was performed on day 2 of life. At 7 months of age, she underwent a repair of her ARM followed 6 months later by closure of her colostomy. With time she became continent of urine, but her bowel habits were variable with some days not opening her bowels at all and other days more than ten times. She was kept on regular anal dilatation, and laxatives were started as she developed a fecal mass. Due to a delay in walking and brisk lower limb reflexes, an MRI of the spine was performed, which revealed a tethered cord. This was operated on when she was 3 years old, to prevent future neurologic impairment and hopefully give her better bowel control. She was often aware of the sensation to defecate but could not control it and so was in nappies even when she started school.

Around the time she was 9 years of age, based on increased diarrhea and failure to gain weight, she was tested for antigliadin antibodies that were found to be positive. A duodenal biopsy revealed villous atrophy suggestive of coeliac disease. Since being on a gluten-free diet, her bowel symptoms have improved dramatically and she is off all laxatives. She is now clean most days with only occasional soiling at night and was able to come out of nappies.

## DISCUSSION

Coeliac disease is common in Caucasians, especially in Western Europe with an incidence of 1:300. Traditionally it was thought to be rare in Asian and African populations but over the past two decades it is being diagnosed more often. Coeliac disease was first diagnosed in India in 1966[1] but since then several large series have been published.[2,3] In people who are genetically predetermined, exposure to gliadin in wheat and barley leads to an autoimmune response targeted against tissue transglutaminase.[4] Antibodies against tissue transglutaminase are the most specific and are recommended for diagnosis. Antigliadin antibodies, as used in our case, are known to have both false-positive and false-negative results. Studies have shown that the genetic markers are similar in India and the West.[5] The small bowel mucosa is predominantly affected with the disease severity fading distally. Involvement can be patchy. Histology reveals villous atrophy with deepening of the crypts of Lieberkuhn with intramucosal lymphocyte infiltration.

Gee first described the classic picture of coeliac disease in children in 1888.[6] He described them as having a pot belly with gluteal and proximal muscle wasting. Due to the mucosal involvement, these children suffered from various degrees of malabsorption, steatorrhea, failure to thrive, and anemia. Due to increased awareness of various disease manifestations, better serologic tests, and easy access to endoscopy, the age at diagnosis and the proportion of children presenting with these classic symptoms has decreased in the West and more and more atypical or silent cases are being diagnosed.[7] However, children in India still present late and almost 80% will have the classic features of diarrhea, anemia, and failure to thrive. The delay in diagnosis is multifactorial. Decreased awareness and availability of serologic tests as well as the presence of other diseases that can present with a similar clinical and histologic picture are partially responsible. Prolonged breast feeding and delay in the introduction of wheat into the diet also accounts for the delay in presentation and diagnosis.

The incidence of ARM is around 1 in 4000 newborns. Almost all children who have been operated on for ARMs will have some degree of functional defecating disorder with the high anomalies being more affected.[8] With an improved understanding of the disease, along with improvement in the surgical approaches, and strict postoperative bowel management, most children can be kept clean and dry. The fecal incontinence in children who had posterior sagittal anorectoplasy for high ARM has been seen to improve at adolescence as their constipation resolves.[9] Those children with refractory incontinence not managed adequately with medical measures are candidates for surgical interventions, which include the antegrade colonic enema and artificial bowel sphincter and gracilis neosphincter construction.

## CONCLUSION

This case demonstrates the rare combination of an Indian child with coeliac disease and ARM. The diagnosis was only made when her bowel symptoms deteriorated and it may be that her fecal incontinence prior to this is partly explained by the unrecognized malabsorption caused by her coeliac disease. Placing her on a gluten-free diet has caused a dramatic improvement to her fecal incontinence with which she had been struggling and had reached the stage of discussion regarding an Antegrade Colonic Enema procedure.

It is important to consider other causes of symptoms in children with chronic disabilities. As is seen in our patient, her symptoms improved markedly and her bowel management became more successful once the diagnosis of coeliac disease was established.
